# *catena*-Poly[[(2,9-dieth­oxy-1,10-phen­anthroline-κ^2^ *N*,*N*′)cadmium(II)]-di-μ-dicyan­amido-κ^4^ *N* ^1^:*N* ^5^]

**DOI:** 10.1107/S1600536808021727

**Published:** 2008-07-19

**Authors:** Xian-Fu Zheng, Cao-Yuan Niu, Cao-Ling Feng, Xin-Sheng Wan, Chun-Hong Kou

**Affiliations:** a College of Sciences, Henan Agricultural University, Zhengzhou 450002, People’s Republic of China

## Abstract

In the title polymer, [Cd(C_2_N_3_)_2_(C_16_H_16_N_2_O_2_)]_*n*_, the Cd^II^ ion is coordinated by two N atoms from one 2,9-dieth­oxy-1,10-phenanthroline mol­ecule and four N atoms from four symmetry-related dicyanamide ions in a distorted octa­hedral geometry. In the 2,9-dieth­oxy-1,10-phenanthroline ligand, the O and C atoms of the eth­oxy groups are located almost in the plane defined by the phenanthroline ring system. Two dicyanamide ions bridge two Cd^II^ ions, which are located on a twofold axis, forming a one-dimensional zigzag chain along the [001] direction. The 2,9-dieth­oxy-1,10-phenanthroline mol­ecules act as bidentate terminal ligands. There are π–π inter­actions between polymeric chains, characterized by a centroid–centroid distance of 3.7624 (2) Å between the phenanthroline rings of two neighbouring chains.

## Related literature

For related literature, see: Brammer (2004 ▶); Chao *et al.* (2000 ▶); Fletcher *et al.* (2001 ▶); Lan *et al.* (2005 ▶); Luo *et al.* (2002 ▶); Jensen *et al.* (2002 ▶); Pijper *et al.* (1984 ▶); Karmakar *et al.* (2006 ▶); Triki *et al.* (2001 ▶); Wang *et al.* (2004 ▶); Bing *et al.* (2004 ▶).
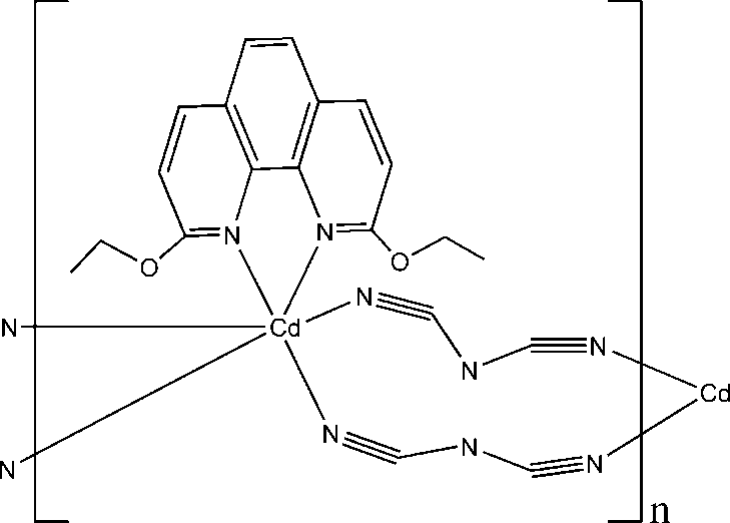


## Experimental

### 

#### Crystal data

[Cd(C_2_N_3_)_2_(C_16_H_16_N_2_O_2_)]
*M* *_r_* = 512.81
Monoclinic, 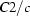

*a* = 14.3605 (9) Å
*b* = 12.4386 (8) Å
*c* = 11.7582 (7) Å
β = 106.676 (1)°
*V* = 2012.0 (2) Å^3^
*Z* = 4
Mo *K*α radiation
μ = 1.12 mm^−1^
*T* = 173 (2) K
0.48 × 0.22 × 0.12 mm

#### Data collection

Siemens SMART CCD area-detector diffractometer
Absorption correction: multi-scan (*SADABS*; Siemens, 1996 ▶) *T*_min_ = 0.615, *T*_max_ = 0.877
6910 measured reflections
2607 independent reflections
2481 reflections with *I* > 2σ(*I*)
*R*_int_ = 0.013

#### Refinement

*R*[*F* ^2^ > 2σ(*F* ^2^)] = 0.019
*wR*(*F* ^2^) = 0.051
*S* = 1.04
2607 reflections
142 parameters
H-atom parameters constrained
Δρ_max_ = 0.35 e Å^−3^
Δρ_min_ = −0.33 e Å^−3^

### 

Data collection: *SMART* (Siemens, 1996 ▶); cell refinement: *SAINT* (Siemens, 1996 ▶); data reduction: *SAINT*; program(s) used to solve structure: *SHELXS97* (Sheldrick, 2008 ▶); program(s) used to refine structure: *SHELXL97* (Sheldrick, 2008 ▶); molecular graphics: *SHELXTL* (Sheldrick, 2008 ▶); software used to prepare material for publication: *SHELXTL*.

## Supplementary Material

Crystal structure: contains datablocks I, global. DOI: 10.1107/S1600536808021727/bh2180sup1.cif

Structure factors: contains datablocks I. DOI: 10.1107/S1600536808021727/bh2180Isup2.hkl

Additional supplementary materials:  crystallographic information; 3D view; checkCIF report

## Figures and Tables

**Table:** 

Cd1—N4	2.2980 (15)
Cd1—N2	2.3580 (16)
Cd1—N1	2.3829 (12)

**Table:** 

N4—Cd1—N4^i^	84.73 (9)
N4—Cd1—N2^i^	86.54 (6)
N4^i^—Cd1—N2^i^	91.14 (6)
N2^i^—Cd1—N2	176.87 (7)
N4—Cd1—N1^i^	168.02 (6)
N2—Cd1—N1^i^	98.21 (5)
N4—Cd1—N1	103.21 (5)
N2—Cd1—N1	84.37 (5)
N1^i^—Cd1—N1	70.51 (6)

Symmetry code: (i) 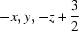.

**Fig. 1.**
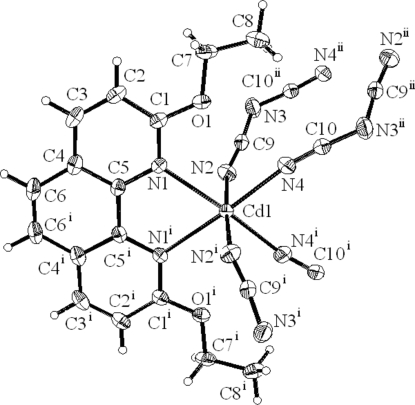
A view of the CdII coordination environment in the polymeric structure of the title compound, showing the atom-labelling scheme. Displacement ellipsoids are drawn at the 50% probability level and H atoms are shown as small spheres of arbitrary radii. Symmetry codes: (i) -x, y, -z + 3/2; (ii) -x, -y + 1, -z + 2.

**Fig. 2.**
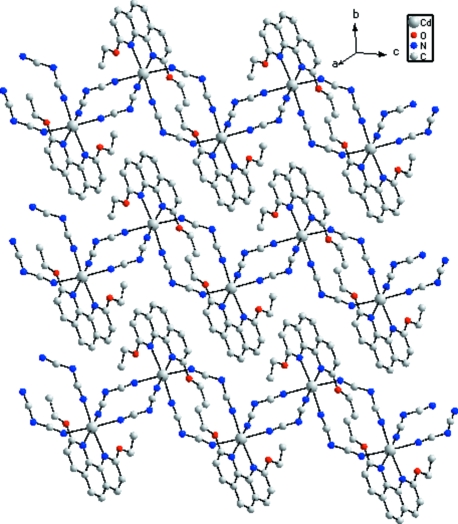
Packing diagram showing the interchain π–π interactions. All H atoms have been omitted for clarity.

## supplementary crystallographic information

### Comment

The construction of novel coordination polymers with the metal-organic frameworks (MOFs) is a very important part of crystal engineering not only for specially functional material purpose but also for their fascinating structures (Fletcher *et al.*, 2001; Brammer, 2004). As the crucial component and also the network spacers of the coordination polymers, organic or inorganic bridging ligands play important roles in the formation of MOFs. Besides, terminal multi-dentate ligands sometimes can tune the network topology constructed by transition metal ions and bridging ligands. So, combining bridging and terminal ligands to synthesize MOFs compounds have made rapid progress recently.

The derivatives of 1,10-phenanthroline are typical terminal bi-dentate organic ligands and also good candidates for building fluorescent compounds, due to the large aromatic ring system (Bing *et al.*, 2004; Chao *et al.*, 2000). Usually, bi-dentate terminal 1,10-phenanthroline ligands occupy certain portions of the metal coordination sphere to prevent the dimensionality of coordination polymers to increase from one-dimensional to two-dimensional or three-dimensional, while the one-dimensional polymer is constructed by other bridging ligands, such as dicyanamide ions (Wang *et al.*, 2004).

Dicyanamide ion is a novel inorganic bridging ligand that has various coordinating modes in the formation of coordination polymers. As an end-to-end single bridge, it can link two metal ions resulting in a one-dimensional chain (Karmakar *et al.*, 2006) or two-dimensional grids (Jensen *et al.*, 2002). One dicyanamide ion can also bridge more than two metal ions through other bridging modes such as *µ*_3_– or *µ*_4_– modes, to afford two-dimensional or three-dimensional multi-dimensional frameworks (Jensen *et al.*, 2002). An end-to-end double bridging mode has also been found in a few cases (Triki *et al.*, 2001). As part of our continuing research of MOFs compounds, we communicate here the crystal structure of the title compound.

In the title compound, the central cadmium ion is in a distorted octahedral coordination environment in which two N atoms from a phenanthroline ring and two N atoms from two symmetry-related dicyanamide ions are located in the equatorial plane, while two N atoms from two distinct dicyanamide ions are in axial positions (Fig. 1). Differences in Cd—N bond lengths, which range from 2.2980 (15) to 2.3829 (12) Å, indicate the distortion from ideal octahedron symmetry around Cd centres. The bond angles around the Cd centre within the range 84.37 (5)–103.21 (5)° for the formally *cis* pair of atoms and 168.02 (6)–176.87 (7)° for the formally *trans* pairs, give further evidences for the distortion (Table 1). The structure of this compound consists of one-dimensional neutral inorganic double chains where two dicyanamide ions act as bridging ligands by their terminal N atoms, bridging two Cd^II^ ions on a twofold axis along the *c* cell axis. Two Cd^II^ ions along the chain are separated by 7.5776 (4) Å. The 2,9-diethoxy-1,10-phenanthroline molecule acts as a bi-dentate terminal ligand.

Two neighbouring infinite one-dimensional chains penetrate into each other through π–π stacking interactions, involving phenanthroline rings, with a centroid to centroid distance of 3.7624 (2) Å and a dihedral angle of 0°. These interactions contribute to the construction of pseudo 2-D layers in the crystal (Fig. 2).

It is noteworthy that only two Cd^II^ coordination compounds constructed using mixed ligands 1,10-phenanthroline and dicyanamide are found in the CSD database. One is a monomer (Lan *et al.*, 2005), composed of two 1,10-phenanthroline molecules and two dicyanamide ions, [Cd(C_2_N_3_)_2_(C_12_H_8_N_2_)_2_], where two dicyanamide ions are in a monodentate coordinating mode and the Cd—*N*(dicyanamide) bond lengths are 2.251 (2) and 2.257 (2) Å. The other, [Cd(C_2_N_3_)_2_(C_12_H_8_N_2_)]_n_, however, is a two-dimensional coordination polymer constructed by one Cd ion, one 1,10-phenanthroline molecule, and two dicyanamide ions in one neutral structural unit (Luo *et al.*, 2002). This compound has a molecular formula similar to that of the title compound, but its two-dimensional layer framework is very different from the one-dimensional chain structure observed in the present structure. The bridging modes of dicyanamide ions tuned by different terminal ligand, 1,10-phenanthroline in the literature (Luo *et al.*, 2002) and 2,9-diethoxy-1,10-phenanthroline in the present work, play a critical role in the structural diversity. The diethoxy groups of 2,9-diethoxy-1,10-phenanthroline can occupy some positions that prevent the linkage of dinuclear subunits Cd-(C_2_N_3_)_2_-Cd by other end-to-end single bridges of dicyanamide ions and the formation of the two-dimensional layered structure. For one terminal ligand molecule, two O and four C atoms of two ethoxy groups are located almost in the plane defined by the phenanthroline ring system. One O and two C atoms of one ethoxy group deviate from the phenanthroline plane by about 0.11, 0.07, and 0.26 Å, respectively.

### Experimental

The organic ligand 2,9-diethoxy-1,10-phenanthroline was prepared according to a reported procedure (Pijper *et al.*, 1984). A solution of NaC_2_N_3_ (0.016 g, 0.2 mmol) in CH_3_OH (10 ml) was carefully layered on a CH_3_OH/CHCl_3_ solution (5 ml/10 ml) of Cd(ClO_4_)_2_.6H_2_O (0.042 g, 0.1 mmol) and 2,9-diethoxy-1,10-phenanthroline (0.024 g, 0.1 mmol) in a straight glass tube. About two weeks later, colourless single crystals suitable for X-ray analysis were obtained. Spectroscopic analysis: IR (KBr, ν cm^-1^): 2990, 2293, 2184, 1603, 1507, 1299, 1041; Analysis: calculated for C_20_H_16_CdN_8_O_2_: C 46.84, H 3.14, N 21.85%; found: C 46.98, H 3.06, N 21.77%.

### Refinement

Carbon-bound H atoms were placed in calculated positions (C—H = 0.95 Å for C2, C3, C6; C—H = 0.99 Å for C7; C—H= 0.98 Å for C8), and were included in the refinement in the riding-model approximation, with *U*_iso_(H) values set at 1.5 times *U*_eq_(C) for methyl H atoms and 1.2 times *U*_eq_(C) for other H atoms. The final difference map had a highest peak at 0.89 Å from atom N3 and a deepest hole at 0.71 Å from atom Cd1, but was otherwise featureless.

### Crystal data

**Table:**

[Cd(C_2_N_3_)_2_(C_16_H_16_N_2_O_2_)]	*Z* = 4
*M**_r_* = 512.81	*F*_000_ = 1024
Monoclinic, *C*2/*c*	*D*_x_ = 1.693 Mg m^−^^3^
Hall symbol: -C 2yc	Mo *K*α radiation λ = 0.71073 Å
*a* = 14.3605 (9) Å	θ = 2.2–29.0º
*b* = 12.4386 (8) Å	µ = 1.12 mm^−^^1^
*c* = 11.7582 (7) Å	*T* = 173 (2) K
β = 106.6760 (10)º	Block, colourless
*V* = 2012.0 (2) Å^3^	0.48 × 0.22 × 0.12 mm

### Data collection

**Table:**

Siemens SMART CCD area-detector diffractometer	2607 independent reflections
Radiation source: fine-focus sealed tube	2481 reflections with *I* > 2σ(*I*)
Monochromator: graphite	*R*_int_ = 0.013
*T* = 173(2) K	θ_max_ = 29.0º
φ and ω scans	θ_min_ = 2.2º
Absorption correction: multi-scan(SADABS; Siemens, 1996)	*h* = −19→18
*T*_min_ = 0.615, *T*_max_ = 0.877	*k* = −14→16
6910 measured reflections	*l* = −15→15

### Refinement

**Table:**

Refinement on *F*^2^	Secondary atom site location: difference Fourier map
Least-squares matrix: full	Hydrogen site location: inferred from neighbouring sites
*R*[*F*^2^ > 2σ(*F*^2^)] = 0.019	H-atom parameters constrained
*wR*(*F*^2^) = 0.051	*w* = 1/[σ^2^(*F*_o_^2^) + (0.0307*P*)^2^ + 0.6141*P*] where *P* = (*F*_o_^2^ + 2*F*_c_^2^)/3
*S* = 1.04	(Δ/σ)_max_ = 0.001
2607 reflections	Δρ_max_ = 0.35 e Å^−^^3^
142 parameters	Δρ_min_ = −0.33 e Å^−^^3^
Primary atom site location: structure-invariant direct methods	Extinction correction: none

### Fractional atomic coordinates and isotropic or equivalent isotropic displacement parameters (Å^2^)

**Table:**

	*x*	*y*	*z*	*U*_iso_*/*U*_eq_	
Cd1	0.0000	0.307814 (10)	0.7500	0.03578 (6)	
N1	0.05844 (9)	0.15137 (10)	0.86539 (10)	0.0365 (2)	
N2	−0.11504 (13)	0.31300 (11)	0.85824 (15)	0.0536 (4)	
N3	−0.13259 (16)	0.38101 (13)	1.04474 (15)	0.0721 (5)	
N4	0.08778 (13)	0.44432 (13)	0.86321 (13)	0.0594 (4)	
O1	0.13134 (9)	0.24924 (10)	1.02404 (9)	0.0502 (3)	
C1	0.11272 (11)	0.15105 (13)	0.97734 (13)	0.0404 (3)	
C2	0.14741 (12)	0.05560 (14)	1.04063 (15)	0.0515 (4)	
H2	0.1877	0.0582	1.1203	0.062*	
C3	0.12161 (13)	−0.03999 (14)	0.98457 (17)	0.0538 (4)	
H3	0.1445	−0.1051	1.0252	0.065*	
C4	0.06132 (12)	−0.04351 (13)	0.86703 (16)	0.0463 (3)	
C5	0.03113 (10)	0.05547 (11)	0.81033 (13)	0.0369 (3)	
C6	0.02939 (14)	−0.14182 (13)	0.80563 (18)	0.0570 (4)	
H6	0.0502	−0.2083	0.8444	0.068*	
C7	0.19439 (13)	0.26036 (16)	1.14355 (14)	0.0510 (4)	
H7A	0.2605	0.2345	1.1481	0.061*	
H7B	0.1691	0.2175	1.1992	0.061*	
C8	0.19725 (16)	0.37679 (18)	1.17551 (16)	0.0649 (5)	
H8A	0.2262	0.4179	1.1231	0.097*	
H8B	0.2365	0.3863	1.2581	0.097*	
H8C	0.1310	0.4025	1.1663	0.097*	
C9	−0.12079 (12)	0.35044 (12)	0.94474 (14)	0.0431 (3)	
C10	0.10564 (12)	0.52463 (13)	0.91050 (14)	0.0458 (3)	

### Atomic displacement parameters (Å^2^)

**Table:**

	*U*^11^	*U*^22^	*U*^33^	*U*^12^	*U*^13^	*U*^23^
Cd1	0.04617 (9)	0.02954 (8)	0.02878 (8)	0.000	0.00622 (6)	0.000
N1	0.0399 (6)	0.0341 (6)	0.0338 (6)	0.0023 (5)	0.0077 (5)	0.0046 (5)
N2	0.0593 (9)	0.0558 (9)	0.0474 (8)	0.0004 (6)	0.0180 (7)	−0.0080 (6)
N3	0.1294 (16)	0.0418 (8)	0.0601 (10)	−0.0173 (9)	0.0510 (11)	−0.0091 (7)
N4	0.0826 (11)	0.0477 (8)	0.0417 (7)	−0.0154 (7)	0.0078 (7)	−0.0055 (6)
O1	0.0608 (7)	0.0476 (7)	0.0316 (5)	0.0014 (5)	−0.0036 (5)	0.0019 (4)
C1	0.0405 (7)	0.0436 (8)	0.0349 (7)	0.0025 (6)	0.0076 (6)	0.0077 (6)
C2	0.0524 (9)	0.0539 (10)	0.0445 (8)	0.0078 (7)	0.0081 (7)	0.0176 (7)
C3	0.0557 (9)	0.0449 (9)	0.0606 (10)	0.0115 (7)	0.0163 (8)	0.0223 (8)
C4	0.0481 (8)	0.0364 (7)	0.0583 (9)	0.0058 (6)	0.0211 (7)	0.0097 (7)
C5	0.0387 (7)	0.0319 (7)	0.0423 (7)	0.0009 (5)	0.0149 (6)	0.0034 (5)
C6	0.0689 (11)	0.0301 (8)	0.0776 (12)	0.0042 (7)	0.0300 (9)	0.0068 (7)
C7	0.0488 (8)	0.0679 (11)	0.0296 (7)	−0.0070 (8)	0.0004 (6)	0.0052 (7)
C8	0.0750 (12)	0.0754 (13)	0.0376 (8)	−0.0053 (10)	0.0056 (8)	−0.0110 (8)
C9	0.0521 (8)	0.0338 (7)	0.0437 (8)	0.0000 (6)	0.0142 (7)	0.0006 (6)
C10	0.0589 (9)	0.0430 (8)	0.0351 (7)	−0.0022 (7)	0.0128 (6)	0.0022 (6)

### Geometric parameters (Å, °)

**Table:**

Cd1—N4	2.2980 (15)	C2—H2	0.9500
Cd1—N4^i^	2.2980 (15)	C3—C4	1.405 (3)
Cd1—N2^i^	2.3580 (16)	C3—H3	0.9500
Cd1—N2	2.3580 (16)	C4—C5	1.408 (2)
Cd1—N1^i^	2.3829 (12)	C4—C6	1.427 (3)
Cd1—N1	2.3829 (12)	C5—C5^i^	1.444 (3)
N1—C1	1.3234 (18)	C6—C6^i^	1.338 (4)
N1—C5	1.3602 (19)	C6—H6	0.9500
N2—C9	1.143 (2)	C7—C8	1.494 (3)
N3—C9	1.292 (2)	C7—H7A	0.9900
N3—C10^ii^	1.299 (2)	C7—H7B	0.9900
N4—C10	1.136 (2)	C8—H8A	0.9800
O1—C1	1.334 (2)	C8—H8B	0.9800
O1—C7	1.4434 (17)	C8—H8C	0.9800
C1—C2	1.414 (2)	C10—N3^ii^	1.299 (2)
C2—C3	1.358 (3)		
			
N4—Cd1—N4^i^	84.73 (9)	C1—C2—H2	120.9
N4—Cd1—N2^i^	86.54 (6)	C2—C3—C4	120.65 (15)
N4^i^—Cd1—N2^i^	91.14 (6)	C2—C3—H3	119.7
N4—Cd1—N2	91.14 (6)	C4—C3—H3	119.7
N4^i^—Cd1—N2	86.54 (6)	C3—C4—C5	117.23 (15)
N2^i^—Cd1—N2	176.87 (7)	C3—C4—C6	122.82 (15)
N4—Cd1—N1^i^	168.02 (6)	C5—C4—C6	119.94 (16)
N4^i^—Cd1—N1^i^	103.21 (5)	N1—C5—C4	122.27 (14)
N2^i^—Cd1—N1^i^	84.37 (5)	N1—C5—C5^i^	118.71 (8)
N2—Cd1—N1^i^	98.21 (5)	C4—C5—C5^i^	119.01 (10)
N4—Cd1—N1	103.21 (5)	C6^i^—C6—C4	121.04 (10)
N4^i^—Cd1—N1	168.02 (6)	C6^i^—C6—H6	119.5
N2^i^—Cd1—N1	98.21 (5)	C4—C6—H6	119.5
N2—Cd1—N1	84.37 (5)	O1—C7—C8	107.58 (15)
N1^i^—Cd1—N1	70.51 (6)	O1—C7—H7A	110.2
C1—N1—C5	118.54 (13)	C8—C7—H7A	110.2
C1—N1—Cd1	125.41 (10)	O1—C7—H7B	110.2
C5—N1—Cd1	116.03 (9)	C8—C7—H7B	110.2
C9—N2—Cd1	137.07 (14)	H7A—C7—H7B	108.5
C9—N3—C10^ii^	122.21 (15)	C7—C8—H8A	109.5
C10—N4—Cd1	160.44 (17)	C7—C8—H8B	109.5
C1—O1—C7	118.95 (13)	H8A—C8—H8B	109.5
N1—C1—O1	113.44 (13)	C7—C8—H8C	109.5
N1—C1—C2	122.96 (15)	H8A—C8—H8C	109.5
O1—C1—C2	123.59 (14)	H8B—C8—H8C	109.5
C3—C2—C1	118.28 (15)	N2—C9—N3	172.45 (18)
C3—C2—H2	120.9	N4—C10—N3^ii^	172.66 (19)
			
N4—Cd1—N1—C1	12.39 (13)	C5—N1—C1—C2	3.1 (2)
N4^i^—Cd1—N1—C1	−118.3 (2)	Cd1—N1—C1—C2	−178.36 (11)
N2^i^—Cd1—N1—C1	100.74 (12)	C7—O1—C1—N1	−176.54 (14)
N2—Cd1—N1—C1	−77.47 (12)	C7—O1—C1—C2	3.4 (2)
N1^i^—Cd1—N1—C1	−178.25 (15)	N1—C1—C2—C3	−1.7 (2)
N4—Cd1—N1—C5	−169.00 (10)	O1—C1—C2—C3	178.35 (15)
N4^i^—Cd1—N1—C5	60.3 (3)	C1—C2—C3—C4	−0.5 (3)
N2^i^—Cd1—N1—C5	−80.65 (10)	C2—C3—C4—C5	1.3 (2)
N2—Cd1—N1—C5	101.15 (10)	C2—C3—C4—C6	−177.76 (17)
N1^i^—Cd1—N1—C5	0.36 (7)	C1—N1—C5—C4	−2.2 (2)
N4—Cd1—N2—C9	−13.0 (2)	Cd1—N1—C5—C4	179.08 (11)
N4^i^—Cd1—N2—C9	−97.7 (2)	C1—N1—C5—C5^i^	177.68 (15)
N1^i^—Cd1—N2—C9	159.45 (19)	Cd1—N1—C5—C5^i^	−1.0 (2)
N1—Cd1—N2—C9	90.1 (2)	C3—C4—C5—N1	0.1 (2)
N4^i^—Cd1—N4—C10	35.8 (4)	C6—C4—C5—N1	179.14 (14)
N2^i^—Cd1—N4—C10	127.3 (4)	C3—C4—C5—C5^i^	−179.80 (16)
N2—Cd1—N4—C10	−50.6 (4)	C6—C4—C5—C5^i^	−0.7 (2)
N1^i^—Cd1—N4—C10	167.9 (3)	C3—C4—C6—C6^i^	178.8 (2)
N1—Cd1—N4—C10	−135.1 (4)	C5—C4—C6—C6^i^	−0.2 (3)
C5—N1—C1—O1	−177.02 (12)	C1—O1—C7—C8	−176.37 (14)
Cd1—N1—C1—O1	1.57 (17)		

Symmetry codes: (i) −*x*, *y*, −*z*+3/2; (ii) −*x*, −*y*+1, −*z*+2.
